# New Appliances and Things Medical

**Published:** 1903-09-19

**Authors:** 


### NEW APPLIANCES AND THINGS MEDICAL.

### THE SCOTTISH WATERPROOF COMPANY, LTD.

(47 COCKBURN STREET, EDINBURGH.)

The above company have sent us a sample of their hygienic leaf-turner—a finger-cap made of indiarubber with a rough surface. This simple contrivance permits of the rapid turning of the leaves of a book, and should altogether do away with the uncleanly process of wetting the finger in the mouth. The price is only twopence, and therefore there is nothing to prevent its being adopted in all offices, libraries, banks, and other places where it must prove very useful.

### PEPSIN AND ZYMINE "PEPULES."

(FAIRCHILD BROS. AND FOSTER, SNOW HILL BUILDINGS, LONDON, E.C.)

This "pepule" is now added to the list of Messrs. Fairchild's excellent digestive preparations. It contains 2 grs. of pepsin and 3 grs. of zymine, the latter containing the three pancreatic enzymes—trypsin, diastase, and steapsin—in active form. In cases where artificial aids to digestion are necessary—and they are only too frequent in these days—this "pepule" should prove of great service, when administered under medical supervision. They are most carefully prepared, easily swallowed, and in every way to be commended. They are supplied in bottles of 25 and 100.

